# Physiologic effects of different hemodynamic patterns of LVAD

**DOI:** 10.3389/fcvm.2025.1645705

**Published:** 2025-08-04

**Authors:** Jiahao Mao, Zhen Gao, Wenyuan Yu, Yang Yu

**Affiliations:** Department of Cardiac Surgery, Beijing Anzhen Hospital, Capital Medical University, Beijing, China

**Keywords:** pulsatile flow, continuous flow, left ventricular assist device (LVAD), heart failure, pulsatility

## Abstract

Left ventricular assist device (LVAD) is an important treatment for patients with end-stage heart failure, which essentially replaces the left ventricle's pumping action to improve peripheral circulation. Its hemodynamic pattern (pulsatile vs. continuous flow) has been a popular research topic in the field. Because of its dependability and endurance, the continuous flow LVAD (CFVAD), as opposed to the first generation of pulsatile flow LVAD (PFVAD), has gained popularity as a mechanical support device in clinical practice in recent years. Many of the complications that arise with CFVAD application are thought to be related to reduced pulsatility. This article provides a review of the physiologic effects of different hemodynamic patterns on the circulatory system and the difference in outcomes of PFVAD vs. CFVAD.

## Introduction

Heart failure is a group of clinical syndromes characterized by ventricular filling or systolic dysfunction due to a variety of causes. Many patients with heart failure progress to advanced heart failure, and they have persistent symptoms with ventricular dysfunction despite optimal medical therapy. Advanced heart failure is considered to have a poor prognosis as well as a high mortality rate of 25%–75% at 1 year (1). The prevalence of heart failure in adults in developed countries is 1%–2% (2). While better medical care extends the survival of people with heart disease, the prevalence of advanced heart failure keeps rising as the population ages. The main treatments for advanced heart failure include heart transplantation and mechanical circulatory assistance. Due to donor scarcity, heart transplantation cannot be a routine treatment. The left ventricular assist device (LVAD) is a commonly deployed mechanical circulatory support device that has been widely used as bridge to transplantation(BTT), bridge to recovery(BTR), bridge to decision(BTD) or destination therapy (DT).

According to hemodynamic characteristics, LVADs are mainly classified into pulsatile-flow and continuous-flow devices. The first generation of LVADs produce pulsatile blood flow by simulating ventricular function. These devices are huge and have a large number of components, including pneumatic or hydraulic actuators, diaphragms and valves. Consequently, they are heavy, power-hungry and prone to malfunctions, whose application is limited by poor device durability (3). Compared to the first generation of pulsatile flow LVAD (PFVAD), continuous flow LVAD (CFVAD) is smaller, technically easier to implant, and have better durability (4). Clinical studies have shown that patients implanted with CFVAD have better clinical survival compared to PFVAD (5). These devices transport blood from the left ventricle to the systemic circulation via centrifugal or axial pumps. However, with the extensive utilization of CFVAD, many complications have arisen, including gastrointestinal bleeding, hemolysis, aortic insufficiency, and pump thrombosis. Recent studies have suggested that the appearance of these complications may be related to the non-physiologic hemodynamic environment generated by CFVAD. Numerous animal experiments and clinical studies have been conducted to compare the effects of PFVAD with CFVAD in terms of hemodynamics, organ perfusion and function, left ventricular unloading, and myocardial remodeling. We think that a discussion of these concerns will assist to study the physiologic role of pulsatile blood flow and encourage further advancements and innovations in LVAD despite the diminishing use of PFVAD. This article provides a review of the physiologic effects and complications of these two LVADs and their associated mechanisms.

## Vascular reactivity to hemodynamics

The physiological effects of pulsatile blood flow on endothelial cells of the vascular wall have been widely demonstrated. Endothelial cells are located between blood flow and vascular tissue, which are directly affected by hemodynamic signals, including shear stress and blood pressure. Shear stress is a fluid velocity-induced tangential force that regulates endothelial cells through mechanical signaling pathways (6). Normal blood flow-induced shear stress can stimulate calcium inward flow and activate signaling pathways linked to the expression of anti-inflammatory transcription factors (7). It also induces increased expression of endothelial nitric oxide synthase (eNOS) and production of endothelium-derived nitric oxide (NO) (8, 9). NO has several anti-atherosclerotic effects, including prevention of oxidative stress, platelet activation, and smooth muscle cell proliferation (10). However, the vascular system is adversely affected by hemodynamic signals that are outside of the physiological range. For example, vascular wall permeability will rise with continuous exposure to low shear conditions, while platelet activation will result with high shear stress (8).

Wang et al. (11) showed that pulsatile blood flow contributes to an increase in NO as well as a decrease in reactive oxygen species (ROS) in vascular endothelial cells, which were mainly consistent with the trend of the dynamic response of intracellular calcium. In heart failure, diminished blood flow pulsatility causes a buildup of ROS in endothelial cells. Ootaki (12) et al. investigated the histologic effects of different levels of pulsatile blood flow on renal arteries via experiments on animals. It was found that only the CFVAD group exhibited extensive hyperplasia of the renal artery wall and activation of the renin-angiotensin system (RAS) in infiltrating inflammatory cells. Similar results were seen in the pulmonary arteries of the calf model (13). In addition to animal models, Haglund (14) suggested using primary human artery endothelial cells from patients older than 55 to create cell culture models and exposed them to different hemodynamic environments. In contrast to pulsatile perfusion, continuous flow perfusion was found to result in up-regulation of endothelin-1(ET-1) and down-regulation of eNOS at the gene level in the ET-1/eNOS signaling pathway, as well as increased transcriptional expression of Nrf-2 and Nrf-2-regulated antioxidant genes; In the meantime, Haglund's group demonstrated that the aforementioned effect was lessened by restoring a specific degree of pulsatility. This shows that vascular endothelial cells may have developed an antioxidant response in response to continuous flow, which was normalized when pulsatility returned.

In summary, pulsatile flow protects the vascular endothelium by sustaining physiological shear stress. CFVAD, on the other hand, may harm the vasculature by activating RAS, triggering an inflammatory response and causing oxidative stress.

## Endothelial function

Flow-mediated dilatation (FMD) is a noninvasive way of evaluating endothelial function in blood arteries. This impact depends on endothelium-derived NO produced by blood flow shear stimulation (15). Amir (16) et al. showed that FMD was significantly lower in patients implanted with CFVADs than in those implanted with PFVADs, which is associated with inferior vascular reactivity; while FMD in the PFVAD group returned to normal levels. Witman et al. (17) found that peripheral vascular endothelial function was significantly impaired in the CFVAD group compared to the control group, including brachial artery pulsatility index as well as standardized FMD. Dlouha's (18) team discovered flow-sensitive microRNAs in the plasma of patients with long-term CFVAD implantation, including a large increase in miR-126, which is negatively related with endothelial dysfunction, and miR-146a, which is implicated in vascular remodeling. It is well known that sympathetic nerve activity regulates arterial blood pressure through baroreceptors. Markham et al. (19) found that muscle sympathetic nerve activity (MSNA) was significantly higher in patients implanted with CFVADs than in patients implanted with PFVADs; while a study by Cornwell et al. (20) found that decreasing CFLVAD pump speed to restore pulsatile flow reduced MSNA and that MSNA was negatively correlated with pulse pressure. These studies suggest that nonpulsatile blood flow reduces the mechanical deformation of baroreceptors, leading to diminished sympathetic inhibition and triggering sustained sympathetic excitation.

Vasoplegic syndrome (VS) is a common surgical complication after in orthotopic heart transplantation (OHT), characterized by low systemic vascular resistance accompanied by low response to vasopressors, resulting in systemic hypotension in the presence of normal cardiac output. Since CFVADs are now the preferred option for BTT in heart transplantation, the incidence of VS following OHT is increasing. Research suggests that CFVAD is an independent predictor of VS (21), possibly due to vascular endothelial dysfunction and adrenal receptor desensitization. In terms of etiology, the release of inflammatory factors after CFVAD implantation stimulates the production of NO by inducible nitric oxide synthase (iNOS), then norepinephrine is partially released with epinephrine to counteract inflammatory vasodilation. Overstimulation of adrenal receptors over time causes receptor desensitization, which predisposes to the production of VS (21).

Overall, CFVAD increases the risk of VS through multiple pathways, including impairment of endothelial function as well as inflammatory responses. Additionally, more high-quality study is required to confirm the independent effects of continuous flow. Correspondingly, pulsatile flow can intermittently increase the shear stress on the vascular wall and protect endothelial function. And it enhances the circulatory system's ability to buffer against changes to arterial flow by maintaining normal stimulation of baroreceptors.

## Hemodynamic changes and left ventricular unloading

Zimpfer et al. (22) reported on 35 cardiac transplant candidates with fixed pulmonary hypertension, all of whom received LVAD implantation as a BTT. Both CFVAD and PFVAD successfully decreased pulmonary vascular resistance after six weeks of follow-up, with no significant difference between the two groups. Similarly, Saidi (23) et al. evaluated the effects of CFVAD vs. PFVAD on pulmonary hemodynamics after 3 months, 1 year, and 3–5 years of implantation. Significant improvements in mean and systolic pulmonary artery pressure were found in all patients, and the effect of PFVAD was more pronounced. The outcomes of two analogous clinical investigations were not equal, which could be attributed to the type of LVAD implanted and the length of follow-up.

In order to simulate acute hemodynamic changes in PFVAD and CFVAD in both healthy and heart failure states, Gohean et al. (24) created computer models and verified them using an animal model of pigs. It was discovered that PFVAD preserved aortic valve flow while maintaining a higher pulse pressure and cardiac output at the same mean flow rate. At the same time, the PFVAD group had lower myocardial work and left atrial pressure, maintaining physiologic blood flow distribution. Such results have been similarly confirmed in other studies (25, 26). Haft et al. (27) evaluated the hemodynamic differences between the two LVADs at 3 months after implantation and found that both LVADs were equal in terms of left ventricular pressure unloading, but the PFVAD was significantly superior than the CFVAD in terms of LV volume unloading; while Garcia et al. (28) found no significant difference between the two LVADs in terms of left ventricular volume and pressure unloading. It is worth noting that the same model of LVAD was used in both experiments, and there was no significant difference in actual flow rates between the two LVADs.

In general, both PFVAD and CFVAD were successful in enhancing cardiac function, lowering pulmonary vascular resistance, and unloading left ventricular pressure in heart failure patients. However, despite controlling for equal or nonsignificant differences in mean flow between the two LVADs, current research is still controversial as to which of them is superior in improving hemodynamics. In clinical applications, the hemodynamic effects of the two LVADs may be altered by a number of factors, including the patient's preoperative baseline level of cardiac function, the LVAD's specific device type and parameters, the measurement method, and the time of installation.

## Left ventricular recovery and remodeling

Chronic heart failure is characterized by a severe impairment in myocardial β-adrenergic receptor (β-AR) signaling, which is caused by G protein-coupled receptor kinase-2 (GRK2)-mediated phosphorylation of β-AR. While inhibition of GRK2 reverses impaired β-AR signaling (29). Akhter et al. (30) investigated 12 patients with heart failure who received CFVAD as BTT and acquired matched left ventricular biopsy specimens at the time of LVAD implantation and heart transplantation. It was found that β-AR signaling was restored to near normal levels after CFVAD support, including the restoration of total β-AR receptor density and the decline in GRK2. However, studies have revealed that PFVAD has a considerably better rate of cardiac recovery than CFVAD. A study by Krabatsch et al. (31) included 387 patients with idiopathic dilative cardiomyopathy implanted with LVADs, using successful LVAD removal and long-term stabilization of cardiac function as the main indicator of myocardial recovery. It was found that the chance for myocardial recovery was three times higher in PFVAD than in CFVAD. Furthermore, a study by Vatta et al. (32) indicated particular damage to the amino-terminus of dystrophin in cardiomyocytes from patients with heart failure, and LVAD treatment was able to restore this damage, with PFVAD superior to CFVAD in dystrophin recovery.

A great deal of biological evidence demonstrates that LVAD restores cardiomyocyte injury and myocardial remodeling in patients with chronic heart failure, including reversing cardiomyocyte hypertrophy and restoring calcium cycling (29). Meanwhile, PFVAD has a better myocardial recovery rate, and more research is still needed to understand its mechanism. Other investigations have demonstrated that CFVAD implantation may cause coronary artery remodeling and fibrosis (33, 34). Ambardekar et al. (33) investigated the effect of CFVAD on coronary artery anatomy in patients with heart failure. It was discovered that compared to the control group, the CFVAD group showed expanded adventitia, breakdown of the internal elastic lamina and more adventitial collagen deposition. Meanwhile vessel density was significantly higher in CFVAD group and positively correlated with the duration of LVAD support. A recent study explored resting myocardial blood flow (MBF) and myocardial flow reserve (MFR) in patients implanted with CFVADs at different pump speed settings (35). The results demonstrated no significant change in MBF at different pump speeds in patients treated with CFVAD, compared to patients with heart failure and the healthy population; at the same time, the high speed group had a lower overall MFR than the low speed group and the heart failure group. Notably, the left anterior descending (LAD) and left circumflex (Lcx) systems revealed a significant reduction in MFR at high pumping speeds, whereas the right coronary MFR remained unchanged. CFVAD-mediated blood flow at high pumping speeds may lack pulsatility, which may result in endothelial dysfunction and increased sympathetic activity, limiting vascular reactivity to metabolic needs. Future studies will examine the effects of LVAD flow patterns on the coronary system by evaluating PFVAD-mediated MBF and MFR under equivalent flow conditions.

## End-organ perfusion and function

Wieselthaler (36) et al. examined endocrine function in nine end-stage heart failure patients with long-term CFVAD implantation. It was discovered that these patients' pituitary hormone response to hypothalamic hormones was identical to that of the healthy population, with the exception of a slightly compromised growth hormone response to growth hormone-releasing hormone; In addition, target glandular hormones such as cortisol, thyroid hormones, and testosterone were secreted normally. Potapov (37) et al. measured serum levels of S-100B (a marker of astrocyte damage) and NSE (a marker of neuronal damage) after PFVAD vs. CFVAD implantation. No significant difference was found between the two groups on biochemical markers of brain injury in the early post-implantation period. Meanwhile, Petrucci et al. (38) found no significant difference in the effects of pulsatile vs. continuous blood flow on neurocognitive function.

Despite variations in cerebral perfusion pressure, cerebral autoregulation maintains relatively constant cerebral blood flow. As previously stated, continuous flow impairs NO generation and causes vascular endothelial dysfunction. NO is a key modulator of cerebral autoregulation, continuous flow may also alter cerebral perfusion (39). Cornwell et al. (40) investigated dynamic cerebral autoregulation by assessing mean arterial pressure and cerebral blood flow velocity in patients receiving LVAD therapy. The findings demonstrated that CFVAD's cerebral autoregulation was not different from that of PFVAD or healthy controls, implying that nonpulsatile blood flow had no effect on cerebral perfusion-related cerebral autoregulation. Nonetheless, a recent investigation verified that metabolism-induced cerebrovascular reactivity (CVR) was influenced by nonphysiologic blood flow (41). To assess CVR under different LVAD blood flow patterns, the team performed a 30-second breath-hold challenge to induce reactive hyperemia in the brain. They discovered that PFVAD-mediated CVR was considerably higher than CFVAD-mediated CVR. Among these, there was a negative correlation between CVR and the pump speed of CFVAD, indicating that metabolism-induced CVR was hampered by non-pulsatile blood flow. The relevant mechanisms still need to be further explored.

Renal insufficiency is common in patients with heart failure and may be linked to renal hypoperfusion. Most renal injuries can be reversed by improved hemodynamics with LVAD. Kamdar (42) et al. retrospectively followed 58 patients with heart failure implanted with LVADs and found that liver and renal function improved and remained in the normal range at 1 and 3 months after implantation, with no significant difference between the CFVAD and PFVAD groups. Nevertheless, it doesn't seem like the improvement in renal function will remain. Brisco (43) et al. included 3,363 adult patients receiving long-term mechanical circulatory support and discovered a considerable increase in eGFR early after LVAD implantation, with a median improvement of 48.9%; However, late eGFR significantly decreased in patients with early improvement in renal function. At one year, eGFR was only 6.7% higher than pre-implantation baseline levels. The mechanisms may include perirenal artery inflammation caused by nonpulsatile blood flow, high central venous pressure from right ventricular failure, and chronic hemolysis linked to LVAD (12, 44). Notably, there was no discernible difference between PFVAD and CFVAD in the incidence of postoperative acute kidney injury and renal function deterioration on long-term support (43, 44). Although nonpulsatile blood flow may potentially impair renal function via vascular remodeling or RAS activation, clinical evidence indicates that blood flow patterns are not a major cause of renal function degradation in LVAD patients.

## Aortic insufficiency

Imamura (45) et al. paired 20 patients who received PFVAD implantation with 20 patients who received CFVAD and followed up for 6 months. The study discovered that the PFVAD group had a higher left ventricular ejection fraction than the CFVAD group, experienced more frequent opening of the native aortic valve, and had a lower incidence of aortic insufficiency. Hatano (46) et al. conducted a retrospective study on 37 patients who received LVADs and had normal aortic valve function prior to implantation. The team assessed the frequency of aortic valve opening and the degree of aortic insufficiency by echocardiography in 28 patients implanted with PFVADs vs. 9 patients implanted with CFVADs, with two in the PFVAD group and seven in the CFVAD group having an appearance of significant aortic insufficiency. Moreover, a multivariate analysis revealed that CFVAD and a decreased preoperative left ventricular ejection fraction were independent risk factors for aortic insufficiency.

Current research reveals that the mechanisms of aortic insufficiency with CFVAD implantation mainly include extended closure of the aortic valve, leading to commissural fusion with myxomatous degeneration of aortic valve leaflets. At the cellular level, the continuous flow of blood resulted in an augmented macrophage response and an increase in the number of valvular interstitial cells. Meanwhile, large-scale protein profiling of aortic valves from patients implanted with CFVAD showed upregulation of TGF-β, which is upregulated in a variety of valve diseases, including aortic stenosis as well as myxomatous mitral valve disease (47). In addition to this, remodeling of the aortic root may also affect aortic regurgitation. Fine (48) et al. conducted a retrospective research that included 162 individuals with chronically implanted CFVADs. Their findings indicated that proximal aortic diameter increased slightly and stabilized in the first 6 months following CFVAD implantation, presumably due to aortic regurgitation.

The preceding research have revealed that PFVAD is more advantageous in maintaining valve function. Maintaining a certain rate of aortic valve opening is critical in lowering the risk of aortic insufficiency. Patients undergoing CFVAD implantation need to be evaluated regularly for valve function and alerted to the potential risk of aortic insufficiency.

## Gastrointestinal bleeding

Crow et al. (49) conducted a retrospective analysis of the rate of gastrointestinal bleeding in 101 patients implanted with LVADs at a single center, 55 with CFVADs and 46 with PFVADs. The findings revealed gastrointestinal bleeding in 12 patients implanted with CFVADs and 3 patients implanted with PFVADs after more than 15 days of implantation. Although mortality was approximate in both groups, the rate of gastrointestinal bleeding was much higher in CFVAD than in PFVAD. The most common cause of bleeding is angiodysplasia (50). Kang (51) et al. discovered that patients receiving CFVAD had dilated, thin-walled vascular structures in the small intestine's submucosa, as well as a considerable increase in vascular density.

It has been shown that CFVAD damages the Von Willebrand factor (VWF), which may be linked to an increased risk of gastrointestinal bleeding (52). The CFVAD uses a centrifugal or axial pump with a high rotating speed to produce a high flow rate of blood, resulting in a high level of shear force. Previous research has shown that the high shear force of CFVAD promotes the proteolytic degradation of high-molecular-weight (HMW) multimers of VWF, which is one of the primary causes of bleeding (53, 54). Vincent (55) et al. studied the association between pulsatility and VWF multimers using LVAD-supported animal models with variations of pulsatility. Animals with low pulsatility levels exhibited faster and more pronounced degradation of VWF multimers, suggesting that pulsatility levels influence the extent of VWF degradation. Meanwhile, Vincent's team discovered that the restoration of pulsatility-induced HMW multimers originated from the release of VWF, stored in the Weibel-Palade bodies (WPBs) of endothelial cells in the vascular wall. It is worth mentioning that the shear force has a positive relationship with the maximum flow rate. The PFVAD produces a higher average shear than the CFVAD while maintaining the same average flow rate. However, the first generation of PFVADs were constrained by their own circumstances and did not achieve the same flow rates as CFVADs, hence the importance of pulsatile perfusion was frequently disregarded. The findings above imply that VWF degradation is connected not only to shear forces, but also to pulsatility provided by LVAD and left ventricle.

## Discussion

PFVAD and CFVAD have become the primary treatment for people with end-stage heart failure. The discrepancies in their physiologic effects and consequences are due to their distinct hemodynamic patterns. PFVAD maintains physiologic blood flow parameters, which help safeguard vascular endothelial function and sustain sympathetic regulation. At the same time, PFVAD has superior myocardial recovery compared to CFVAD. First-generation pulsatile LVADs are prone to catastrophic failure modes, including fatigue-induced diaphragm rupture and progressive bearing wear resulting in sudden pump arrest, characterized by high infection rates and low clinical survival rates. While axial continuous-flow LVADs demonstrate greater durability with miniaturized implantation, specific problems exist, such as pump thrombosis, gastrointestinal bleeding and cerebrovascular accidents. The centrifugal flow-pump HeartMate 3 is the most recent advancement in CFVAD. Its advantages include a fully magnetically levitated pump that eliminates mechanical wear and heat generation, as well as wide blood flow gaps that reduce shear stress, effectively reducing the incidence of pump thrombosis and improving associated clinical outcomes. Axial-flow and centrifugal-flow pumps can greatly improve peripheral perfusion because of their high flow rates. It may instead lead to unique complications such as vascular endothelial dysfunction, acquired VWF deficiency and aortic insufficiency. LVAD significantly improves survival and quality of life in patients with end-stage heart failure through mechanical unloading, but multiple challenges remain regarding myocardial recovery. Pulsatility has been proven in studies to be an independent predictor of myocardial recovery, and the molecular biological mechanisms of pulsatile flow remain to be further explored. Future prospective multicenter studies are required to evaluate the benefits of PFVAD in myocardial recovery and to provide a uniform removal assessment procedure of LVAD.

Researchers have devoted themselves to merging the advantages of the two types of LVAD device by developing pulsatility control algorithms and examining flow modulation strategies to generate pulsatility in centrifugal CFVADs. For example, the HeartMate 3 has an "artificial pulse mode" (56), where the pump speed is adjusted periodically to superimpose pulsatile flow on continuous flow. Lower aortic valve opening is frequently associated with CFVAD, which is typically caused by insufficient pressure gradients between the left ventricle and aorta as a result of extreme left ventricular pressure unloading. It has been argued that keeping some of the ejection function of the patient's own heart is desirable, and that the pulsatility produced by the native heart interacts synergistically with the CFVAD to improve pulsatility. This approach is based on the patient's cardiac function and LVAD flow matching. These methods are theorized to help with long-term support for LVADs. Long-term follow-up studies are still needed to determine whether adding pulsatility to CFVAD improves clinical outcomes.

The current study suggests that perfusion of vital organs varies according to hemodynamic effects of LVAD, including decreased metabolism-induced CVR due to nonphysiologic blood flow and impaired MFR in the left coronary artery system due to CFVAD's high pump rate. This could be linked to the sensitivity of nonpulsatile blood flow to cause vascular endothelial dysfunction. However, there is currently a gap in studies comparing PFVAD to CFVAD in essential organ perfusion. When comparing the physiological effects of the traditional PFVAD to those of the modern CFVAD, experimental designs frequently fail to effectively control variables due to multivariate interactions and technological limitations, affecting the analysis of causal relevance of study findings. Notably, percutaneous left ventricular assist devices (pLVADs) are the most recent advancement in LVAD device technology. They are implanted through a less invasive channel (e.g., the femoral artery) rather than an open thorax, dramatically reducing surgical trauma. The realization of different blood flow patterns by pump speed modulation in the same pLVAD may theoretically aid to manage the variables and realize future study in this field of issues.
